# Gene signatures associated with prognosis and chemotherapy resistance in glioblastoma treated with temozolomide

**DOI:** 10.3389/fgene.2023.1320789

**Published:** 2023-12-18

**Authors:** Tonia Carter, Robert K. Valenzuela, Srinivasulu Yerukala Sathipati, Rafael Medina-Flores

**Affiliations:** ^1^ Center for Precision Medicine Research, Marshfield Clinic Research Institute, Marshfield, WI, United States; ^2^ Department of Pathology (Neuropathology), Marshfield Clinic, Marshfield, WI, United States

**Keywords:** chemotherapy resistance, glioblastoma, oncogene, somatic variant, temozolomide

## Abstract

**Background:** Glioblastoma (GBM) prognosis remains extremely poor despite standard treatment that includes temozolomide (TMZ) chemotherapy. To discover new GBM drug targets and biomarkers, genes signatures associated with survival and TMZ resistance in GBM patients treated with TMZ were identified.

**Methods:** GBM cases in The Cancer Genome Atlas who received TMZ (*n* = 221) were stratified into subgroups that differed by median overall survival (mOS) using network-based stratification to cluster patients whose somatic mutations affected genes in similar modules of a gene interaction network. Gene signatures formed from differentially mutated genes in the subgroup with the longest mOS were used to confirm their association with survival and TMZ resistance in independent datasets. Somatic mutations in these genes also were assessed for an association with OS in an independent group of 37 GBM cases.

**Results:** Among the four subgroups identified, subgroup four (*n* = 71 subjects) exhibited the longest mOS at 18.3 months (95% confidence interval: 16.2, 34.1; *p* = 0.0324). Subsets of the 86 genes that were differentially mutated in this subgroup formed 20-gene and 8-gene signatures that predicted OS in two independent datasets (Spearman’s rho of 0.64 and 0.58 between actual and predicted OS; *p* < 0.001). Patients with mutations in five of the 86 genes had longer OS in a small, independent sample of 37 GBM cases, but this association did not reach statistical significance (*p* = 0.07). Thirty-one of the 86 genes formed signatures that distinguished TMZ-resistant GBM samples from controls in three independent datasets (area under the curve ≥ 0.75). The prognostic and TMZ-resistance signatures had eight genes in common (*ANG*, *BACH1*, *CDKN2C*, *HMGA1*, *IFI16*, *PADI4*, *SDF4*, and *TP53INP1*). The latter three genes have not been associated with GBM previously.

**Conclusion:** *PADI4*, *SDF4*, and *TP53INP1* are novel therapy and biomarker candidates for GBM. Further investigation of their oncologic functions may provide new insight into GBM treatment resistance mechanisms.

## 1 Introduction

Glioblastoma (GBM) has a low median overall survival (mOS) of approximately 15 months even with the standard first-line therapy of surgery followed by radiotherapy and temozolomide (TMZ) chemotherapy (Stupp et al., 2005). This treatment is not curative as the tumor eventually reoccurs (Wen et al., 2020). TMZ’s cytotoxicity is due to its modification of genomic DNA to generate O^6^-methylguanine, which is mispaired with thymine during DNA replication (Newlands et al., 1997). Repetitive futile cycles of the mismatch repair pathway to repair the mismatched base pair produce DNA strand breaks, inducing cell cycle arrest and eventually leading to apoptosis (Karran et al., 1993; Newlands et al., 1997). A DNA repair enzyme encoded by the O^6^-methylguanine-DNA methyltransferase (*MGMT*) gene repairs O^6^-methylguanine by removing the TMZ-added methyl group, counteracting the cytotoxic effects of TMZ (Singh et al., 2021). The epigenetic silencing of *MGMT* by the methylation of CpG sites in its promoter is associated with longer OS in GBM patients treated with TMZ (Hegi et al., 2005), and *MGMT* promoter methylation status is both a known prognostic biomarker in GBM and a predictive biomarker of response to TMZ treatment (Weller et al., 2009). *MGMT* promoter methylation is present in more than 90% of GBM patients who have longer-term survival compared with approximately 30% of all GBM patients (Stupp et al., 2014). However, the signaling pathways that control *MGMT* activity in GBM are incompletely understood, and GBM has a dismal prognosis irrespective of the *MGMT* promoter methylation status (Wick et al., 2014). Sustained efforts have been made to develop and test new drugs for GBM, but GBM has remained refractory to most new therapies (Khasraw et al., 2022; van Solinge et al., 2022).

The identification of new drug targets has the potential to increase therapeutic options that improve outcomes in GBM. GBM tumor progression is promoted by the genetically heterogeneous nature of the tumor (Brennan et al., 2013), the invasive growth of tumor cells (Venkataramani et al., 2022), the low immunogenicity of the tumor (Pearson et al., 2020), and mechanisms of treatment resistance (Singh et al., 2021). Taken together, these characteristics draw attention to the biological complexity of treatment response in GBM. To begin to identify prospective drug targets for GBM, this hypothesis-generating study aimed to uncover gene signatures associated with survival in GBM patients who received TMZ because the genes that make up those signatures have the potential to provide insight into the numerous biological pathways that influence patient outcomes, facilitate the discovery of targetable components within those pathways, and also serve as prognostic biomarkers for GBM. The first step of the approach was to stratify GBM patients into subgroups that differed by mOS using network-based stratification (NBS) (Hofree et al., 2013), an algorithm that clusters together patients with genetic variants in similar regions of a gene interaction network. Next, the genes that were differentially mutated in the subgroup(s) with a mOS longer than that reported for standard TMZ chemotherapy were used to determine biological pathways associated with survival. Finally, the group of differentially mutated genes was used to identify gene signatures associated with OS and TMZ resistance in independent GBM datasets.

## 2 Materials and methods

### 2.1 Study subjects, samples, and datasets

To identify gene networks associated with mOS after TMZ treatment, public data on simple somatic variants and clinical characteristics from The Cancer Genome Atlas (TCGA) were analyzed for the 221 GBM cases (out of a total of 606 TCGA GBM cases) who had received concomitant and/or adjuvant TMZ chemotherapy and had data available on OS and simple somatic variants from primary tumor (the numbers of included and excluded subjects are shown in Supplementary Figure S1). The simple somatic variants included single nucleotide variants and small insertions/deletions and multiple base substitutions ≤ 200 base pairs. Datasets GSE108474 and GSE7696 from the Gene Expression Omnibus data repository were used to confirm the association between the mutated genes and OS. Tumor microarray gene expression data were available for 81 GBM patients who received TMZ in the GSE108474 dataset (Gusev et al., 2018) and 43 GBM patients who received TMZ in the GSE7696 dataset (Murat et al., 2008). To determine whether any of the genes that were correlated with OS were also mutated in an independent group of GBM cases treated with TMZ, somatic variant data were generated from the treatment-naïve, primary tumors of 37 patients (out of a total of 118 patients) who were diagnosed with GBM from 2004 to 2012 at Marshfield Clinic Health Systems (MCHS) in Marshfield, Wisconsin, and had received concomitant and/or adjuvant TMZ chemotherapy (Carter et al., 2018). Of the 118 patients, DNA could be extracted from the archived tumor specimens of 74 patients. Exome sequencing could be performed successfully on 37 of the 74 DNA samples. To determine whether the genes correlated with OS could also discriminate between TMZ-resistant and control GBM cell lines or tumors, gene expression data in the GSE151680, GSE193957, and GSE145128 datasets were analyzed. GSE151680 had RNA-seq data for three TMZ-resistant and three control samples for each of two GBM cell lines (U87 and U251); GSE193957 had microarray data for three TMZ-resistant and three control samples from the U87 cell line (Choo et al., 2023); GSE145128 had microarray data for treatment-naïve tumor and the matched tumor that recurred after TMZ treatment in seven GBM patients (Kebir et al., 2023).

### 2.2 Network-based stratification analysis

NBS, performed using pyNBS (Huang et al., 2018), was used to generate somatic mutation profiles from TCGA GBM somatic variant data and stratify those profiles into subgroups associated with OS. The NBS algorithm uses network propagation to integrate the somatic mutation profiles with a gene interaction network and non-negative matrix factorization to cluster the integrated profiles into a pre-determined number of groups from *k* = 2 up to *k* = 12. For each pre-determined number of clusters, network propagation and non-negative matrix factorization were applied to 1,000 different subsets of the TCGA GBM somatic variant data, with each subset containing 80% of the patients and 80% of the mutated genes sampled at random without replacement, followed by consensus clustering of the aggregated results of the 1,000 subsets into a single cluster result. The STRING database (version 11.5) (Szklarczyk et al., 2023) of known and predicted protein-protein interactions was the source of the human gene interaction network for NBS. Only interactions having a confidence score > 0.7 (indicating high-confidence interactions; 16,795 nodes and 252,013 edges out of 19,385 nodes and 5,969,249 edges) were input to NBS. The protein nodes represented 16,127 genes. The alpha tuning parameter, which controls the distance that a mutation signal can diffuse through the gene network during propagation, was set to 0.7 for network propagation using the STRING network, as recommended in the original NBS report. Default settings were accepted for other NBS parameters. Kaplan-Meier analysis and the log rank test (Schober and Vetter, 2018) were implemented in pyNBS to generate survival curves for each of the NBS subgroups within *k* consensus clusters and determine whether NBS-assigned clusters are associated with OS. Cox proportional hazards regression (Cox, 1972) was also performed to assess whether NBS subgroups were associated with OS independent of other clinical factors known to predict survival in GBM, including age at diagnosis (years), type of surgery at initial pathologic diagnosis (biopsy/tumor resection/other), and radiation therapy (yes/no/unknown).

### 2.3 Identification of differentially mutated genes in NBS subgroups

To identify the gene interaction network regions that contribute the most to distinguishing the somatic mutation profiles of tumors in different NBS subgroups (*k* = 4 clusters), the non-parametric Significance Analysis of Microarrays (SAM) (Tusher et al., 2001) method was applied to the integrated mutation profile (consisting of 16,127 genes) that was generated after network propagation, as described in the original NBS report. Each subgroup, in turn, was compared with all other subgroups to detect genes with significantly different network-smoothed mutation states in that subgroup. SAM (version 5.0) was run using the SAMR shiny app with data type chosen as array - two class unpaired, median center the arrays as “yes”, statistical test as Wilcoxon rank sum test, number of permutations as 1,000, and random seed as the default value. The threshold for statistical significance was determined by the value of a tuning parameter, delta, which is user-selected based on the false discovery rate. A fold-change ≥ 2 and false discovery rate < 0.05 were selected for this analysis.

### 2.4 Gene Ontology term enrichment

To determine the Gene Ontology (GO) Biological Process categories that were over- or under-represented among a set of genes compared to a reference list of genes, enrichment analysis was performed directly from the GO website (Gene Ontology, 2021), with the use of the PANTHER over-representation test (Mi et al., 2019b) from the PANTHER gene classification resource (Mi et al., 2019a). The GO database version used was DOI: 10.5281/zenodo.6799722, released 1 July 2022, and the list of background genes comprised the 16,127 genes that formed the human gene interaction network for NBS analysis. The *p*-values were calculated by Fisher’s exact test with false discovery rate correction, and a corrected *p*-value < 0.05 was considered statistically significant.

### 2.5 Predicting survival and differentiating between temozolomide-resistant and control samples

We utilized the least absolute shrinkage and selection operator (LASSO) (Tibshirani, 1996) technique to estimate the OS of GBM patients. LASSO employs L1 regularization, a process that facilitates the regularization of certain coefficients that contribute to the output evaluation. This regularization technique effectively aids in the feature selection process. For the determination of the tuning parameter λ, we selected the minimum λ after conducting 100 iterations of 10-fold cross-validation (10-CV). In assessing the performance of the model, we employed Spearman’s rank correlation coefficient ρ) (Schober et al., 2018) and the mean absolute error (Willmott and Matsuura, 2005) as measurement metrics.

For the prediction of TMZ resistance in patients with GBM, we employed several standard machine learning methods available in Weka (Hall et al., 2009), including Naïve Bayes, simple logistic, sequential minimal optimization, random forest, and J48. The assessment of these methods was conducted based on evaluation metrics such as accuracy, sensitivity, specificity, and area under the receiver operating characteristic curve (AUC).

### 2.6 Tumor DNA extraction

For MCHS patients, GBM tumor DNA was extracted from formalin-fixed and paraffin-embedded (FFPE) tissue for exome sequencing. A matched germline DNA sample was not available. Sections with a thickness of 10 µM were cut from each tissue block and, after discarding the first three sections, three to four sections, depending on tissue size, were sampled in triplicate and placed in micro-centrifuge tubes for DNA extraction. Tissues were extracted within 12 h of cutting. DNA extractions were performed using the GeneRead™ DNA FFPE kit (Qiagen, Valencia, CA) according to the manufacturer’s recommendations. The quality (260/280 ratio) of each DNA sample was assessed using a NanoDrop™ spectrophotometer (ThermoFisher Scientific, Waltham, MA). Initial DNA quantity was determined using either a BR (broad range) or HS (high sensitivity) kit on a Qubit 2 Fluorometer (ThermoFisher Scientific). DNA integrity (fragmentation) and final concentration were determined with Genomic DNA ScreenTape on a TapeStation 2,200 (Agilent Technologies, Santa Clara, CA) prior to sample pooling. DNA from triplicate samples were pooled as necessary to obtain the appropriate concentration and volume for downstream testing.

### 2.7 Exome sequencing

DNA library preparation and exome sequencing were performed by Admera Health (South Plainfield, NJ). DNA sample quality was assessed by Agilent DNA 6000 Nano Reagent on an Agilent 2,100 Bioanalyzer (Agilent Technologies, Santa Clara, CA) and quantified by Qubit DNA HS assay (ThermoFisher Scientific). Library preparation for exome sequencing was performed with KAPA HyperPrep kits (Roche) and IDT xGen indexes (Illumina Inc., San Diego, CA) following the manufacturer’s instructions. Samples were pooled and sequenced on an Illumina NovaSeq S4 sequencer for 150 base pairs read length in paired-end mode, with an output of 260 million reads per sample.

### 2.8 Exome sequence data processing

The quality of sequencing reads were checked with FastQC (version 0.11.2) before the reads were aligned to a human genome reference sequence (GRCh38. d1. vd1) using the Burrows-Wheeler Aligner (version 0.7.5a) (Li and Durbin, 2009). Duplicate reads generated by polymerase chain reaction (PCR) were marked and removed using MarkDuplicates in Picard (version 1.73), and local realignment of reads followed by base quality recalibration were performed using the Genome Analysis Toolkit (GATK version 4.1.4.1) (Van der Auwera et al., 2013). Variants were called from recalibrated reads using the Mutect2 algorithm in GATK (McKenna et al., 2010). To improve the quality of variant calls, variants derived from normal tissue, known as a Panel of Normals, was employed as a reference by Mutect2 to detect sequencing and alignment artifacts (Cibulskis et al., 2013). The Panel of Normals for a tumor-only variant calling pipeline, consisting of variants from approximately 5,000 TCGA exome sequencing samples of individuals without cancer, was downloaded from the online Genomic Data Commons data portal for input to Mutect2 (Zhang et al., 2021). The CollectSequencingArtifactMetrics and FilterByOrientationBias commands in GATK were used to filter called variants for sequence artifacts that arise from the deamination of cytosines by formaldehyde in FFPE tissue or the oxidation of guanine to 8-oxoguanine (Costello et al., 2013). Next, PureCN (version 1.22.2) was used to classify variants by somatic/germline status based on an assessment of tumor purity, ploidy, loss of heterozygosity, and copy number (Oh et al., 2020). Variants that were flagged as unreliable or were assigned a somatic posterior probability of less than 0.8 by PureCN were removed. Variants not located in the target region or supported by fewer than five reads were also removed. Next, filtered variants were annotated using Variant Effect Predictor (version 105) (McLaren et al., 2016). Only rare variants, defined as having an alternate allele frequency < 1% in the GnomAD database (Karczewski et al., 2020), were considered as somatic variants.

### 2.9 *MGMT* promoter methylation

DNA methylation assays were performed by EpigenDx (Hopkinton, MA) using their ASY470-FS2 protocol, which involved bisulfite sequencing of eight CpG sites in the differentially methylated region 2 (Malley et al., 2011) of the *MGMT* promoter. DNA was available from 31 of the 37 MCHS patients to perform the assays. For each patient, 300 ng of GBM tumor DNA was bisulfite treated using the EZ DNA Methylation kit (Zymo Research, Inc., Irvine, CA). Bisulfite-treated DNA was purified according to the manufacturer’s protocol and eluted to a final volume of 46 μL. PCRs were performed using 1 μL of bisulfite treated DNA and 0.2 μM of each primer. One primer was biotin-labeled and HPLC purified in order to purify the final PCR product using sepharose beads. PCR product was bound to Streptavidin Sepharose High Performance beads (GE Healthcare Life Sciences), after which the immobilized PCR products were purified, washed, denatured with a 0.2 μM NaOH solution, and rewashed using the Pyrosequencing Vacuum Prep Tool (Pyrosequencing, Qiagen), as per the manufacturer’s protocol. Next, 0.5 μM of sequencing primer was annealed to the purified single-stranded PCR products. 10 μL of the PCR products were sequenced by pyrosequencing on the PSQ96 HS System (Pyrosequencing, Qiagen), following the manufacturer’s instructions. The methylation status of each CpG site was determined individually as an artificial C/T single nucleotide polymorphism using QCpG software (Pyrosequencing, Qiagen). The methylation level at each CpG site was calculated as the percentage of the methylated alleles divided by the sum of all methylated and unmethylated alleles. The mean methylation level was calculated using methylation levels of all measured CpG sites within the targeted region. Each experiment included non-CpG cytosines as internal controls to detect incomplete bisulfite conversion of the input DNA. In addition, a series of unmethylated and methylated DNA were included as controls in each PCR. PCR bias testing was performed by mixing unmethylated control DNA with *in vitro* methylated DNA at different ratios (0%, 5%, 10%, 25%, 50%, 75%, and 100%), followed by bisulfite modification, PCR, and pyrosequencing analysis. A mean methylation level > 7% was used as the cutoff for scoring samples as unmethylated or methylated (Kristensen et al., 2016).

### 2.10 Statistical methods

The median and 95% confidence interval for OS and time to progression were determined by Kaplan-Meier analysis, and comparisons of OS or time to progression among subgroups were performed using the log rank test, which generated *p*-values for the comparisons (Schober and Vetter, 2018). Multivariate analyses of OS were performed using Cox proportional hazard regression (Cox, 1972). The correlation between OS and time to progression and between actual and predicted OS was determined by Spearman’s rank correlation coefficient (Spearman’s ρ) (Schober et al., 2018). The test statistic for Spearman’s rank correlation coefficient was calculated as:

$$t=\frac{r\sqrt{n-2}}{\sqrt{1-r^{2}}}$$

with *r* being the sample correlation coefficient and *n* being the number of subjects with no missing data for the pair of variables (Kendall and Gibbons, 1990). The two-tailed *p*-value is 2 x P (T > t) where T follows a Student’s t distribution with *n* – 2 degrees of freedom. The proportion of subjects with a methylated *MGMT* promoter was compared among the subgroups in the TCGA dataset using the Chi-squared test and between the two subgroups in the smaller, independent dataset of 37 GBM cases using Fisher’s exact test. Because this was a hypothesis-generating study, *p*-values were not used for hypothesis testing but for identifying genes that warrant further exploration as GBM biomarkers or drug targets. The results and *p*-values are to be considered explorative, and no threshold level of statistical significance should be fixed. Correction for multiple testing was not performed. Survival analysis, Cox proportional hazards regression, Spearman’s rank correlation, the Chi-squared test, and Fisher’s exact test were performed using the R software program (R-Core, 2021).

In LASSO models (Tibshirani, 1996) used for predicting OS based on gene signatures, mean absolute error was used as a measure of the average magnitude of the error produced by a LASSO model. Mean absolute error (Willmott and Matsuura, 2005) was determined by calculating the magnitude of the difference between predicted OS and observed OS for each subject (absolute error for each subject) and taking the average of the absolute errors for the entire group of subjects. Therefore, the lower the value of mean absolute error for a LASSO model, the smaller the differences between OS predicted by the model and observed OS. To assess the performance of machine learning methods to classify TMZ resistant and control samples using gene signatures, values of accuracy, sensitivity, specificity, and the AUC were determined for each machine learning method (Kourou et al., 2015). Accuracy indicated the proportion of correct predictions made by a machine learning method and was calculated as the number of correct predictions/total number of predictions. Sensitivity measured how well a machine learning method could correctly identify TMZ resistant samples and was calculated as True Positives/(True Positives + False Negatives). Specificity measured how well a machine learning method could correctly identify control samples and was calculated as True Negatives/(True Negatives + False Positives). The AUC measured the ability of a machine learning method to distinguish between TMZ resistant and control samples. True positives were TMZ resistant samples that were correctly predicted to be TMZ resistant; false positives were control samples that were falsely predicted to be TMZ resistant; true negatives were control samples that were correctly predicted to be controls; false negatives were TMZ resistant samples that were falsely predicted to be controls.

## 3 Results

### 3.1 Glioblastoma stratification by somatic mutation profiles and identification of genes associated with survival

**FIGURE 1:**
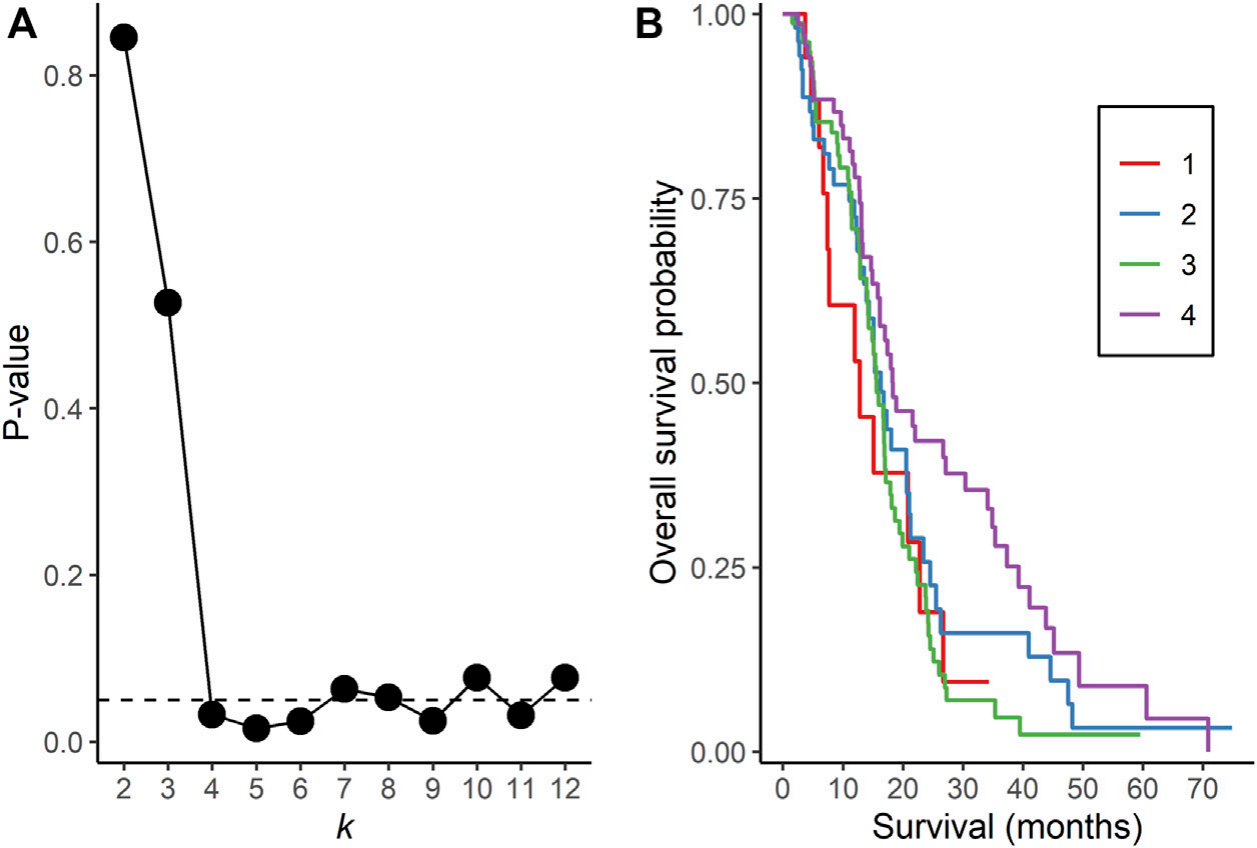
Network Based Stratification analysis of TCGA GBM tumors. **(A)** *p*-values from the log rank test comparing overall survival time between assigned subgroups for cluster sizes *k* = 2 to 12. The horizontal, dotted line represents a *p*-value of 0.05. **(B)** Kaplan-Meier curves of overall survival in each subgroup for *k* = 4 clusters.

NBS analysis of somatic mutation data for the 221 TCGA GBM tumors indicated that *k* = 4 or more clusters were associated with OS based on the log rank test (Figure 1A), and further analyses to identify the gene interaction subnetworks of relevance to OS in TMZ-treated GBM were conducted using the NBS subgroups within *k* = 4 clusters. mOS, as determined by Kaplan-Meier analysis, was highest in subgroup 4 and lowest in subgroup 1 (*p* = 0.0324; Table 1; Figure 1B). Cox proportional hazards regression using data for all 221 subjects showed that subgroups 1 and 3 (compared with subgroup 4 as a reference) were associated with an increased risk of death, independent of other known clinical prognostic factors (Table 2). When the regression was restricted to the 206 subjects without *IDH1/2* variants, similar results were observed (Table 2). Time to progression after initial treatment, an important indicator of GBM treatment efficacy, and thus, of TMZ resistance (Han et al., 2014), was also compared among the four subgroups. Data were available on time to progression for 215 subjects (17, 51, 78, and 68 in subgroups 1, 2, 3, and 4, respectively), and time to progression was positively correlated with overall survival (Spearman’s rank correlation = 0.60, *p* < 3.15 × 10^−22^; Supplementary Figure S2). The median and 95% confidence interval for time to progression was 6.0 (3.5, 7.7) months for subgroup 1, 7.5 (6.0, 9.2) months for subgroup 2, 5.5 (4.9, 6.9) months for subgroup 3, and 7.1 (5.7, 9.3) months for subgroup 4, as determined by Kaplan-Meier analysis. *p* = 0.0103 for the comparison based on the log-rank test, indicating that time to progression was significantly different among the four subgroups, with subgroups 1 and 3 having a shorter median time to progression (suggestive of greater TMZ resistance) than subgroup 4.

**TABLE 1:** Overall survival (months) based on Kaplan-Meier analysis of four TCGA GBM subgroups as assigned by NBS.

Subgroup	*N*	Number of deaths	Median overall survival (95% confidence interval)	*P* ^a^
1	17	12	12.8 (7.5, NA)	0.0324
2	54	39	16.3 (14.0, 21.3)	
3	79	59	15.6 (14.1, 17.9)	
4	71	47	18.3 (16.2, 34.1)	

NA, not available.

^a^ Comparison among all four groups using the log-rank test.

**TABLE 2:** Cox proportional hazards regression of TCGA GBM.

Characteristic	All subjects (*N* = 221)		Subjects without *IDH1/2* variants (*N* = 206)	
	Odds ratio (95% confidence interval)	*P*	Odds ratio (95% confidence interval)	*P*
NBS subgroup				
4	Reference	1.0000	Reference	1.0000
1	2.05 (1.07, 3.93)	0.0308	2.06 (1.04, 4.09)	0.0379
2	1.23 (0.80, 1.89)	0.3531	1.18 (0.76, 1.83)	0.4609
3	1.60 (1.07, 2.37)	0.0205	1.54 (1.02, 2.31)	0.0380
Age at diagnosis (years)	1.02 (1.01, 1.03)	0.0068	1.02 (1.00, 1.03)	0.0564
Type of surgery at initial diagnosis				
Biopsy	Reference	1.0000	Reference	1.0000
Resection	1.19 (0.78, 1.80)	0.4291	1.21 (0.79, 1.86)	0.3890
Other	12.06 (1.48, 98.12)	0.0200	13.83 (1.68, 114.09)	0.0147
Radiation therapy				
Yes	Reference	1.0000	Reference	1.0000
No	2.70 (0.81, 9.02)	0.1056	2.81 (0.84, 9.23)	0.0948
Unknown	0.35 (0.05, 2.54)	0.3003	0.33 (0.05, 2.37)	0.2686

Fifty (22.6%) of the 221 subjects had missing data on *MGMT* methylation status; therefore, this variable was not included as a covariate in the regression model. Of the 171 subjects with *MGMT* methylation status available, 7 (46.7%) of 15 in subgroup 1, 18 (43.9%) of 41 in subgroup 2, 24 (37.5%) of 64 in subgroup 3, and 34 (66.7%) of 51 in subgroup 4 had a methylated *MGMT* promoter (*p* = 0.0169; Chi-squared test). Therefore, each subgroup had a mixture of subjects with and without *MGMT* promoter methylation, and the subgroup with the longest mOS had a greater proportion of subjects with a methylated *MGMT* promoter. Differentially mutated genes numbered 6,683, 447, 507, and 86 genes in subgroups 1, 2, 3, and 4, respectively, and GO enrichment analyses of these genes identified statistically significant biological process terms that varied by subgroup (Supplementary Tables S1–4).

### 3.2 Gene Ontology analysis and validation of association with overall survival using gene signatures

**FIGURE 2:**
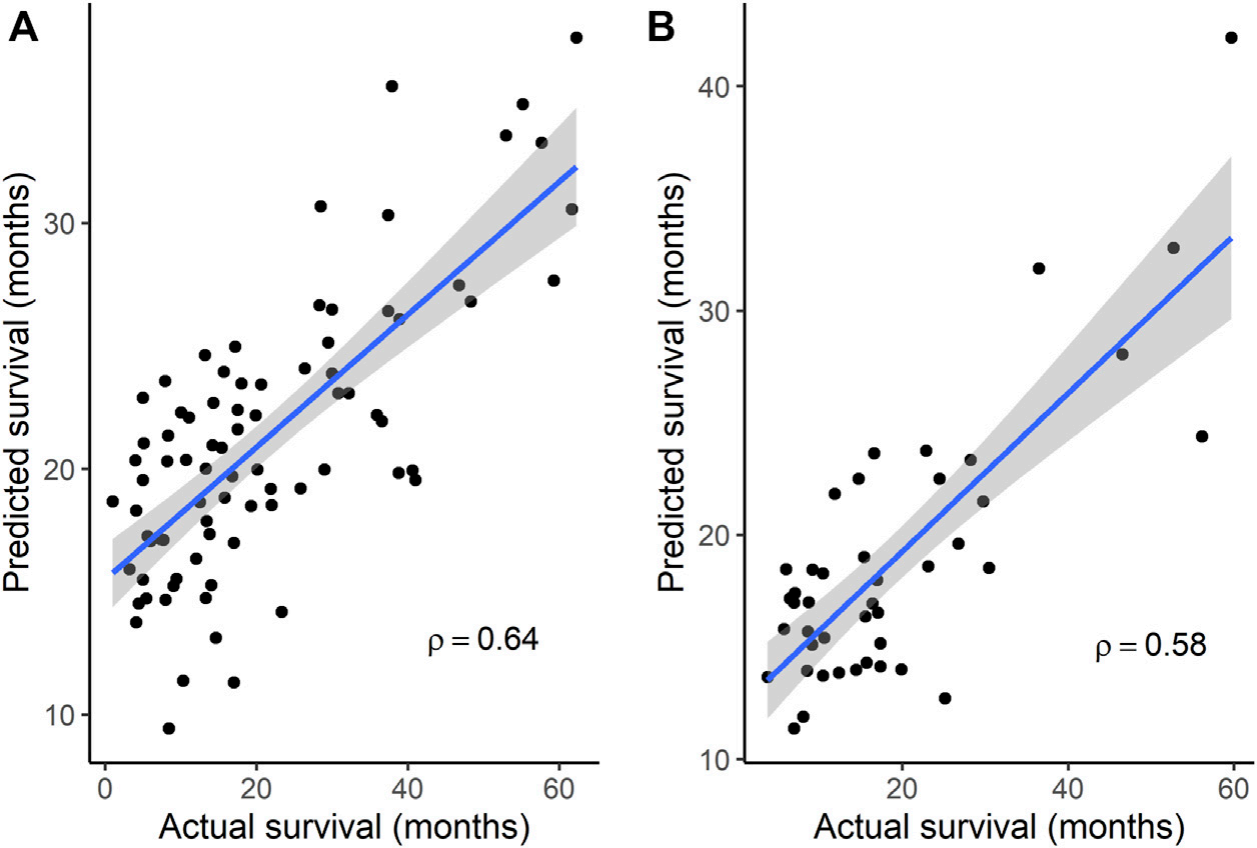
Correlation between actual survival and survival predicted from gene signatures. **(A)** A 20-gene signature was used to predict survival in data set GSE108474 (*N* = 81). *p* = 8.83 × 10^−11^ for Spearman’s ρ. **(B)** An 8-gene signature was used to predict survival in data set GSE7696 (*N* = 43). *p* = 4.84 × 10^−5^ for Spearman’s ρ. In each plot, the line through the points is the linear regression line, and the shading represents the 95% confidence region for the regression line.

Because subgroup 4 had a mOS > 15 months, which is the mOS reported for TMZ-treated GBM patients, and also was significantly longer than the mOS of subgroups 1 and 3, additional analyses focused on subgroup 4. Statistically significant terms from GO enrichment analysis (Supplementary Table S4) for the 86 differentially mutated genes of this subgroup (Supplementary Table S5) were related to the regulation of intrinsic apoptotic signaling by a *TP53* mediator or in response to DNA damage, the regulation of cyclin-dependent protein serine/threonine kinase activity, DNA damage checkpoint signaling, the negative regulation of transcription, cellular senescence, the regulation of cysteine-type endopeptidase activity in apoptosis, and the positive regulation of release of cytochrome c from mitochondria. To determine whether gene signatures associated with OS in independent groups of GBM patients could be identified from among the 86 genes, microarray gene expression data from the GSE108474 and GSE7696 datasets and exome sequence data from the 37 GBM tumors of MCHS patients were analyzed. In the GSE108474 dataset, 20 genes (represented by 24 probes) out of 86 were selected as features that could predict survival in the 81 TMZ-treated GBM patients (Supplementary Table S6). In the GSE7696 dataset, 8 genes (represented by 10 probes) out of 86 were selected as features that could predict survival in the 43 TMZ-treated GBM patients (Supplementary Table S6). The gene signatures from the two datasets had the *HMGA1* gene in common. Spearman’s rank correlation between actual and predicted OS was 0.64 (*p* = 8.83 × 10^−11^) and 0.58 (*p* = 4.84 × 10^−5^) in the GSE108474 and GSE7696 datasets, respectively (Figures 2A, B).

### 3.3 Somatic mutations and overall survival in an independent sample

**FIGURE 3:**
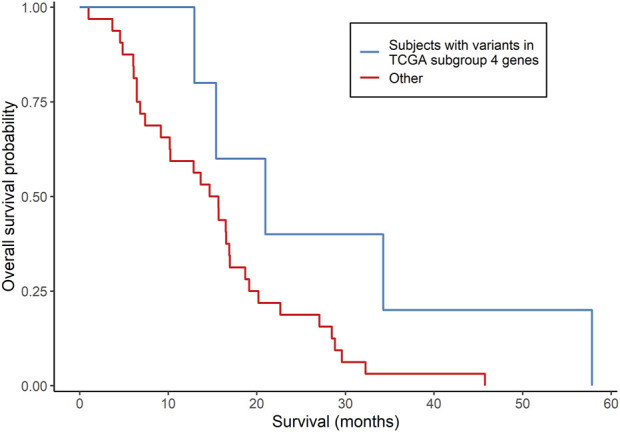
Comparison of survival according to subgroups defined by somatically mutated genes in an independent group of GBM patients. Kaplan-Meier analysis compared subjects who had somatic variants in genes that were differentially mutated in TCGA subgroup 4 (*N* = 5) with all other subjects (*N* = 32). *p* = 0.0697 from the log-rank test.

The clinical characteristics of the 37 MCHS patients are shown in Supplementary Table S7. All 37 patients started, and 34 of the patients completed, concomitant TMZ and/or at least one cycle of adjuvant TMZ. mOS (95% confidence interval), representing the length of time between date of surgery for GBM and the date of death or end of study follow-up (31 December 2015), was 15.7 (12.9, 19.1) months (Supplementary Table S3), as determined by Kaplan-Meier analysis. Because the tumors were archived clinical specimens, a matched specimen of normal DNA was unavailable, and a tumor-only bioinformatics procedure was used to call somatic variants. The mean sequencing depth was between 30 X and 103 X for 28 tumors and between 14 X and 30 X for the other nine tumors. A total of 999 rare, somatic single nucleotide variants or small insertions/deletions in 905 genes were called (Supplementary Table S8), with an average of 27 (range of 1–64) variants detected per tumor. Three hundred and thirty-four (33.4%) of the variants have been reported previously in the COSMIC database of somatic mutations (version 96) (Tate et al., 2019) and, in 21 tumors, 30 variants were detected in 26 genes reported to be mutated recurrently in GBM (Brennan et al., 2013; Frattini et al., 2013). Five patients had five variants in four (*FUBP1*, *L3MBTL1*, *LCOR*, and *USP42*) of the 86 genes that were differentially mutated in TCGA GBM subgroup 4. Three (*FUBP1*, *L3MBTL1*, and *USP42*) of the four genes were among the 20 genes predictive of OS in the GSE108474 dataset. Similar to TCGA GBM subgroup 4, these five patients had a higher mOS than the other 32 patients (21.0 *versus* 15.3 months, respectively), but this difference did not reach statistical significance (*p* = 0.0697; log-rank test; Figure 3). No subjects had *IDH1/2* variants. The *MGMT* promoter was methylated in two (40.0%) of the five patients and 18 (56.3%) of the other 32 patients (*p* = 0.4174; Fisher’s exact test). Overall, the results indicated that gene signatures selected from the 86 genes were associated with GBM survival in two of the three independent datasets.

### 3.4 Identification of overlapping genes between signatures of survival and of temozolomide resistance

**FIGURE 4:**
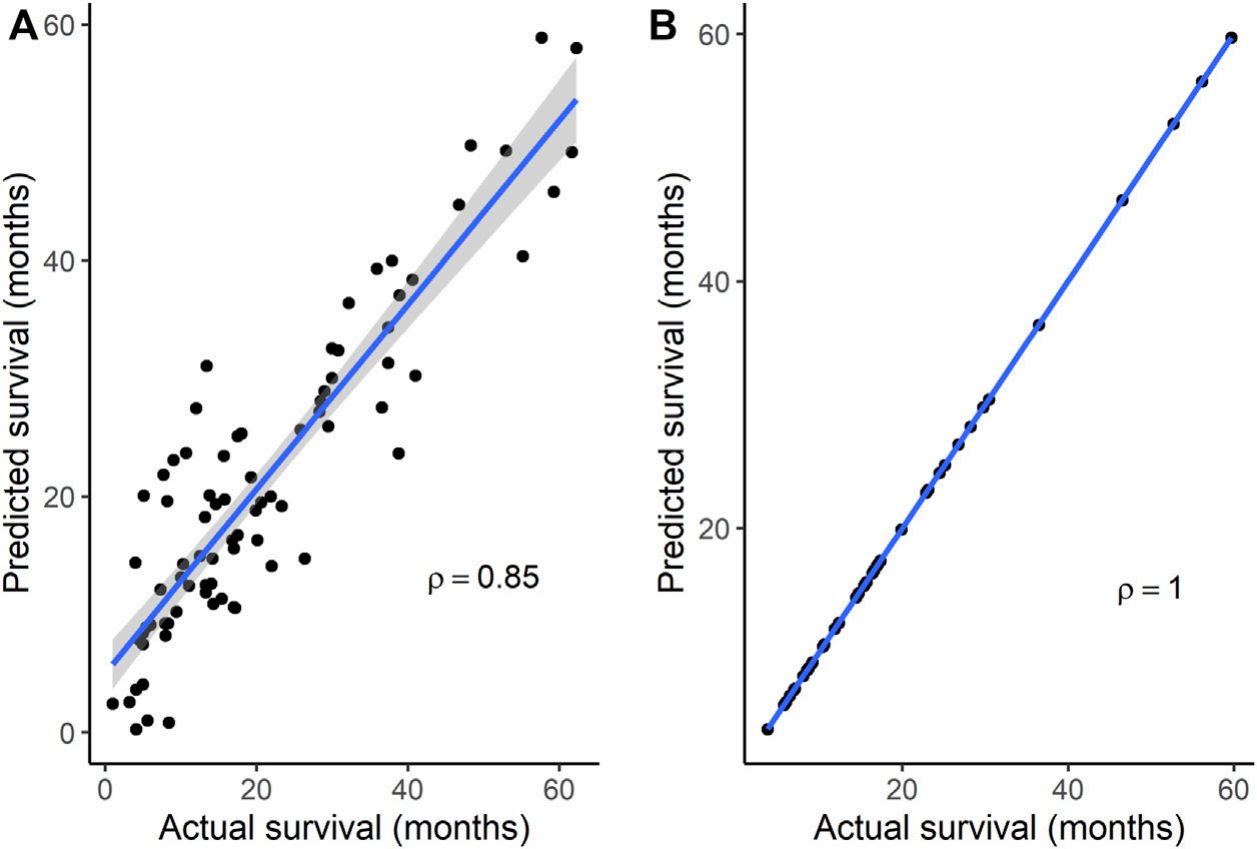
Correlation between actual and predicted survival using a combination of genes in signatures that could distinguish TMZ-resistant and control samples. Survival was predicted for datasets **(A)** GSE108474 (*N* = 81) and **(B)** GSE7696 (*N* = 43). *p* < 0.0001 for Spearman’s ρ in each dataset. In each plot, the line through the points is the linear regression line, and the shading represents the 95% confidence region for the regression line.

To identify gene signatures associated with TMZ resistance, five prediction algorithms were applied to expression data for the 86 genes from the GSE151680, GSE193957, and GSE145128 datasets. For the GSE151680 and GSE193957 data, the AUC was ≥ 0.75 for all five algorithms, whereas, for GSE145128 data, the AUC was ≥ 0.75 only for the Naïve Bayes and random forest algorithms (Table 3). Feature selection identified signatures consisting of 11 genes in GSE151680 data, 11 genes (represented by 12 probes) in GSE193957 data, and 15 genes (represented by 15 probes) in GSE145128 data that could distinguish between TMZ-resistant and control GBM samples (Supplementary Table S9). Six genes (*DNAJC9*, *HIPK4*, *PCBP4*, *PPP1R13L*, *SCAF8*, and *USP11*) overlapped between pairs of the three gene signatures. To determine whether the three gene signatures could also predict GBM survival, each signature, separately and also combined into one group of 31 unique genes, was used to predict OS in the GSE108474 and GSE7696 datasets. The 66 unique probes that tagged the 31 genes in these two datasets were used as features for prediction. Prediction with each separate gene signature resulted in a moderate correlation between actual and predicted OS for the two datasets, with slightly higher correlation and lower mean absolute error values for the GSE7696 dataset (Table 4). The correlation increased to 0.85 (Figure 4A) and 1.00 (Figure 4B) for datasets GSE108474 and GSE7696, respectively, when the combined group of 31 genes was used for prediction. The eight genes that overlapped between the set of 31 genes that could distinguish TMZ-resistant from control GBM samples and the set of 27 genes in the two gene signatures predictive of OS in the GSE108474 and GSE7696 datasets were *ANG*, *BACH1*, *CDKN2C*, *HMGA1*, *IFI16*, *PADI4*, *SDF4*, and *TP53INP1*. Thus, these were genes in common between signatures of prognosis and of TMZ resistance.

**TABLE 3:** Discrimination between TMZ-resistant and control GBM samples using gene signatures in three independent datasets.

Dataset	Method	Accuracy (%)	Sensitivity	Specificity	Area under the curve
GSE151680	Naïve Bayes	83.3	0.75	1.00	0.97
	Simple logistic	83.3	0.83	0.83	0.91
	Sequential minimal optimization	100.0	1.00	1.00	1.00
	Random forest	91.7	1.00	0.85	0.94
	J48	75.0	080	0.74	0.75
GSE193957	Naïve Bayes	100.0	1.00	1.00	1.00
	Simple logistic	100.0	1.00	1.00	1.00
	Sequential minimal optimization	100.0	1.00	1.00	1.00
	Random forest	100.0	1.00	1.00	1.00
	J48	83.3	1.00	0.75	0.83
GSE145128	Naïve Bayes	78.6	0.83	0.75	0.79
	Simple logistic	50.0	0.50	0.50	0.51
	Sequential minimal optimization	71.4	0.71	0.71	0.71
	Random forest	78.6	0.75	0.83	0.84
	J48	35.7	0.37	0.33	0.33

**TABLE 4:** Performance of TMZ-resistance gene signatures in predicting GBM overall survival.

Dataset for survival prediction	Dataset used to select gene signature	Spearman’s rank correlation between actual and predicted survival		Mean absolute error
		ρ	*P*	
GSE108474	GSE151680	0.45	2.82 x 10^-5^	10.7
GSE108474	GSE193957	0.52	7.92 x 10^-7^	11.1
GSE108474	GSE145128	0.46	1.22 x 10^-5^	10.4
GSE7696	GSE151680	0.53	0.00027	7.9
GSE7696	GSE193957	0.74	1.35 x 10^-8^	4.6
GSE7696	GSE145128	0.67	8.06 x 10^-7^	5.9

Of the eight genes, three (*PADI4*, *SDF4*, and *TP53INP1*) have not been investigated previously for associations with GBM. Two *PADI4* variants were detected in subgroups 1 and one *PADI4* variant was detected in subgroup 4 (Supplementary Table S10). A *SDF4* variant was detected in subgroup 1 and a *TP53INP1* variant was detected in subgroup 4. Each of the variants in the three genes was present in a single tumor; therefore, the frequency of each variant in the GBM sample was 0.45% (1/221). The frequency of *PADI4* variants was 1.36% (3/221). Two of the *PADI4* variants and the *TP53INP1* variant have been detected in other cancer types in the COSMIC database (Supplementary Table S10). The *PADI4* p. Ser496Phe variant was detected in one sample of skin carcinoma, the *PADI4* p. Phe602 synonymous variant in two samples of endometrial cancer and one sample of malignant melanoma, and the *TP53INP1* p. Ile66Val variant in one sample of carcinoma of the bile duct. Somatic variants in the three genes have also been reported in several other cancer types in TCGA, with the percentage of cases having a variant ranging from 0.20% to 7.87%, depending on the cancer type (Supplementary Table S11).

## 4 Discussion

Genes that were differentially mutated in the subgroup with the longest mOS, compared with the other subgroups, were enriched in pathways related to intrinsic apoptosis signaling, cell cycle signaling, the DNA damage response, and cellular senescence, all mechanisms that affect GBM tumor growth and TMZ resistance. Intrinsic apoptosis signaling is initiated by the tumor suppressor p53 (*TP53*) in response to an intracellular stress signal such as DNA damage, with subsequent activation of a caspase cascade leading to apoptotic cell death (Li et al., 1997; Tomita et al., 2006). *TP53* also regulates the expression of genes, including cyclins and cyclin-dependent kinases, necessary for progression through the cell cycle (Engeland, 2018). Somatic variants in *TP53* are frequently detected in GBM (Brennan et al., 2013), and TMZ induces cell cycle arrest and senescence in GBM in a *TP53*-dependent manner (Hirose et al., 2001). The PI3K/AKT/mTOR pathway, which also regulates the cell cycle and promotes cell proliferation and survival (Porta et al., 2014), has a mutation in at least one pathway gene in > 80% of GBM patients, causing constitutive activation and aberrant function of the pathway (Cancer Genome Atlas Research, 2008). Furthermore, *AKT* activation protects against the cytotoxic effects of TMZ in a GBM cell line (Hirose et al., 2005). The activation of DNA damage response upstream regulators, the serine/threonine kinases *ATR* and *ATM*, is associated with a reduced load of TMZ-induced DNA double strand breaks and increased TMZ resistance in GBM (Eich et al., 2013), as are mutations in *MSH6*, a mismatch repair gene (Yip et al., 2009). Cellular senescence, characterized by stable cell cycle arrest triggered by damaging stress signals, can exert context-dependent anti- or pro-tumorigenic effects on GBM (Beltzig et al., 2022; Salam et al., 2023). The GBM tumor growth arrest associated with cellular senescence induced by TMZ can be considered anti-tumorigenic (Hirose et al., 2001). In a report describing a pro-tumorigenic effect, tumor microvascular endothelial cells that had been irradiated after removal from GBM tumors were shown to be senescent and TMZ-resistant while also being able to support the proliferation of GBM stem cells (Borovski et al., 2013).

Therapeutic agents that target these pathways are the subject of recently completed and ongoing GBM clinical trials (Wen et al., 2020). Drugs that target the interaction of p53 protein with the E3 ubiquitin ligase, *MDM2*, seek to relieve the inhibition of p53 by *MDM2* and rescue p53 transcriptional activation of pro-apoptotic factors such as *BAX* and *BBC3* (Puca et al., 2009). GBM showed no response to everolimus (Chinnaiyan et al., 2018), which targets mTOR, but other drugs that target mTORC1/2 and cyclin-dependent kinases in the cell cycle machinery are under evaluation (Wen et al., 2020). Inhibitors of the DNA damage response are also being evaluated because DNA repair is partly responsible for TMZ resistance in GBM (Wen et al., 2020). Anti-senescence agents are of interest in GBM because senescent cells are therapy-resistant and can be the precursors of a relapsed tumor (Beltzig et al., 2022). A suggested, two-step strategy involves the use of a chemotherapy drug to induce tumor cell senescence followed by the use of a senolytic to ablate the senescent cells before the tumor progresses and reoccurs (Chojak et al., 2023). TMZ is a potent inducer of senescence (Hirose et al., 2001), and pre-clinical studies of glioma cells have identified other inducers to be nutlin-3a (Villalonga-Planells et al., 2011) and resveratrol (Filippi-Chiela et al., 2013), among others, and senolytics to include BH3 mimetics that target pro-survival *BCL2* family members, which are regulators of apoptosis (Schwarzenbach et al., 2021). The failure of most experimental drugs to extend GBM survival emphasizes the therapeutic challenges in GBM, among which are the genetic heterogeneity within and between tumors, redundant signaling pathways, and gaps in knowledge regarding the contributions of genetic mutations to tumor growth and treatment resistance (Wen et al., 2020).

The identification of gene signatures predictive of OS in independent datasets in this study confirmed the association of GBM survival with the 86 differentially mutated genes in subgroup 4. Subsets of the 86 genes also formed gene signatures that could distinguish TMZ-resistant from control GBM samples, and the pool of 31 genes in these signatures were also predictive of OS, suggesting that some of the genes potentially can function as biomarkers of both prognosis and response to TMZ treatment. To minimize reliance on findings specific to individual datasets, the overlap between signatures of prognosis and TMZ resistance was determined, resulting in eight overlapping genes being identified.

Five (*ANG, BACH1, CDKN2C, HMGA1, IFI16*) of the eight genes have shown associations with GBM in other reports. The expression of angiogenin (*ANG*), a potent stimulator of angiogenesis, was inversely correlated with survival in GBM patients, and *Ang* deficiency prolonged the survival of mice with platelet derived growth factor-induced GBM (Yang et al., 2022). The GBM tumor in these mice showed lower proliferation, less invasiveness, reduced angiogenesis, and higher apoptosis compared with control mice. *ANG* knockdown also decreased cell proliferation and increased apoptosis in GBM cell lines (Yang et al., 2022). The inhibition of *ANG* with neomycin, a small molecule that prevents binding of *ANG* to its receptor, *PLXNB2*, decreased GBM growth in mice with platelet derived growth factor-induced GBM and a xenograft mouse model, demonstrating the potential of *ANG* inhibitors in GBM therapy (Yang et al., 2022). High expression of *BACH1*, a transcription factor, has been detected in GBM (Yuan et al., 2022). *BACH1* overexpression enhanced the expression of *MGMT* leading to TMZ resistance in a GBM cell line with a hypomethylated *MGMT* promoter and a xenograft mouse model, whereas *BACH1* depletion sensitized TMZ-resistant cells to TMZ (Nie et al., 2016). These effects were blocked by *TP53*, which inhibits *MGMT* by preventing the binding of *SP1* to the *MGMT* promoter, but the antagonistic effects of *TP53* could be overcome by *BACH1* overexpression (Nie et al., 2016). The low expression of *BACH1* in combination with wild-type *TP53* was associated with longer survival in patients who received TMZ, suggesting that the targeting of this pathway holds potential for regulating TMZ resistance (Nie et al., 2016). Co-deletion of *CDKN2C*, a cyclin-dependent kinase inhibitor and tumor suppressor, and *CDKN2A* in GBM cells predicted sensitivity to palbociclib (PD0332991), a selective *CDK4/6* inhibitor that is being evaluated in an ongoing GBM clinical trial (NCT03158389) (Wiedemeyer et al., 2010; Cen et al., 2012). *HMGA1*, a gene that alters chromatin architecture to regulate transcription, is highly expressed in some GBM stem cell lines and its silencing reduces the self-renewal and sphere-forming efficiency of these cells and sensitizes them to TMZ (Colamaio et al., 2016). Moreover, decreased *HMGA1* expression in a xenograft mouse model, resulting from knockdown of an upstream long non-coding RNA regulator, correlated with delayed GBM tumor growth and increased survival (Mineo et al., 2016). The binding of *IFI16*, a sensor of DNA in the innate immune response, to *ARPC1B*, a subunit of the actin-related protein-2/3 complex, resulted in activation of the NF-kappa-B pathway that contributed to promotion of a mesenchymal phenotype transformation and radiotherapy resistance in GBM stem cells and a xenograft mouse model (Gao et al., 2022). This study further showed that GBM cell lines with high expression of *ARPC1B* and *IFI16* were more sensitive to ceralasertib (AZD6738), an inhibitor of *ATR*. *IFI16* was also upregulated in GBM cells treated with both Y15 (a small molecule inhibitor of activated focal adhesion kinase) and TMZ (Huang et al., 2014), a combination that decreases viability and tumor growth in GBM cells (Golubovskaya et al., 2013).

The functions of three (*PADI4*, *SDF4*, *TP53INP1*) of the eight genes in GBM remain to be explored. *PADI4*, an enzyme that converts arginine residues to citrulline resulting in recognition of the citrullinated proteins by the immune system, is highly expressed in GBM (Sase et al., 2017; Neira et al., 2022). However, reports regarding the role of *PADI4* in cancer are conflicting. Some studies reported high *PADI4* expression in other cancers (Liu et al., 2019) and showed that *PADI4* overexpression is associated with pro-tumorigenic properties such as increased tumor cell proliferation, migration, clone forming ability, and metastasis, and reduced apoptosis (Chang et al., 2022). Other studies observed low *PADI4* expression in cancer (Indeglia et al., 2023) and found that *PADI4* can suppress tumor cell growth (Tanikawa et al., 2009). *PADI4* is also suggested to function as a tumor suppressor that interacts with p53 to regulate the transcription of p53 target genes (Indeglia et al., 2023). The potential of *PADI4* to serve as a drug target in cancer was demonstrated by the finding that a small molecule inhibitor of *PADI4* activated *SESN2* and other p53 target genes leading to mTORC1 signaling pathway inhibition, perturbation of autophagy, and suppression of tumor cell growth (Wang et al., 2012). In addition, the use of citrullinated proteins as epitopes in cancer immunotherapy is an area of active research (Brentville et al., 2020). The role of *SDF4*, a calcium-binding protein that regulates calcium-dependent cellular activities, in GBM has not been described, but in other cancers, *SDF4* is highly expressed and promotes tumor growth, migration, and distant metastasis (Luo et al., 2016). *SDF4* expression was elevated in a colorectal cancer cell line (Ji et al., 2009) and in pancreatic cancer cells compared with non-neoplastic pancreatic ductal cells (Gronborg et al., 2006). A long non-coding RNA, *LINC00173*, that is upregulated in nasopharyngeal cancer cells, could promote the growth, migration and metastasis of nasopharyngeal cancer cells by interacting with *RAB1B* to facilitate *SDF4* secretion in a *RAB1B*-dependent manner (He et al., 2023). The reversal of *LINC00173*-mediated cancer cell progression by *SDF4* knockdown suggested the potential of the *LINC00173*-*RAB1B*-*SDF4* pathway to be a drug target for nasopharyngeal cancer. The overexpression of *TP53INP1*, a tumor suppressor that promotes the transcriptional activity of *TP53* on its target genes, induces G1 cell cycle arrest and enhances *TP53*-mediated apoptosis in cancer cells (Tomasini et al., 2003). *TP53INP1* also interacts directly with components of the autophagy pathway to induce autophagy-dependent cell death (Seillier et al., 2012). In pediatric ependymoma, a malignant glial cell tumor, high expression of the microRNA, miR-124-3p, and low expression of one of its target genes, *TP53INP1*, correlated with shorter progression-free survival, suggesting the potential to target miR-124-3p and *TP53INP1* in new therapeutic approaches for this cancer (Margolin-Miller et al., 2017). The involvement of *TP53INP1* in GBM tumor growth and therapy resistance needs further study.

A study limitation was the small sample sizes of the GEO independent datasets, which lessened the statistical power for detecting correlations between actual and predicted OS and distinguishing TMZ-resistant from control samples. The small size of the MCHS sample also diminished power to detect a significant difference in mOS between study groups. No matched germline DNA sample was available for this dataset, prompting the use of a PureCN tumor-only bioinformatics workflow that can accurately classify variants as somatic *versus* germline. However, the low depth of exome sequencing coverage of some of the MCHS samples likely hampered the detection of somatic variants in these samples. Another limitation was uncertainty about whether time to progression in TCGA GBM data was based on true disease progression that was documented by imaging according to RANO criteria (Wen et al., 2010).

## 5 Conclusion

In conclusion, targeting the molecular events at the basis of GBM holds potential for the development of new therapeutic strategies in GBM. Somatically mutated genes associated with mOS after TMZ treatment function in the known oncogenic pathways of apoptosis, cell cycle control, the DNA damage response, and cellular senescence. Subsets of these genes form signatures of GBM prognosis or TMZ resistance, and the overlap of these subsets has identified genes that may influence both TMZ resistance and patient survival in GBM. *PADI4*, *SDF4*, and *TP53INP1* have been little studied in relation to GBM and are new GBM gene candidates. Further investigation is needed to evaluate whether these genes are useful for developing interventions to improve the anticancer activity of TMZ and can serve as drug targets or predictive biomarkers of response to GBM treatment. Additional studies should also explore the molecular mechanisms through which somatic mutations in these genes have an impact on the transition of initially chemotherapy-sensitive tumors to TMZ-resistant tumors.

## Data Availability

The datasets for this article are not publicly available due to concerns regarding participant/patient anonymity. Requests to access the datasets should be directed to the corresponding author.
