# Supplementary material for: Gene signatures associated with prognosis and chemotherapy resistance in glioblastoma treated with temozolomide
Source: Front Genet. 2023 Dec 18;14:1320789. doi: 10.3389/fgene.2023.1320789 (PMC10802164; doi:10.3389/fgene.2023.1320789)
Supplement: Supplementary file 4 [file DataSheet3.DOCX]

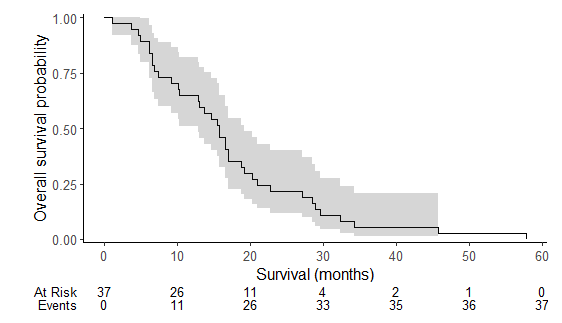


Figure S3. Overall survival in 37 subjects diagnosed with glioblastoma at Marshfield Clinic. The shaded area is the 95% confidence interval.
