# Supplementary material for: Gene signatures associated with prognosis and chemotherapy resistance in glioblastoma treated with temozolomide
Source: Front Genet. 2023 Dec 18;14:1320789. doi: 10.3389/fgene.2023.1320789 (PMC10802164; doi:10.3389/fgene.2023.1320789)
Supplement: Supplementary file 9 [file DataSheet2.DOCX]

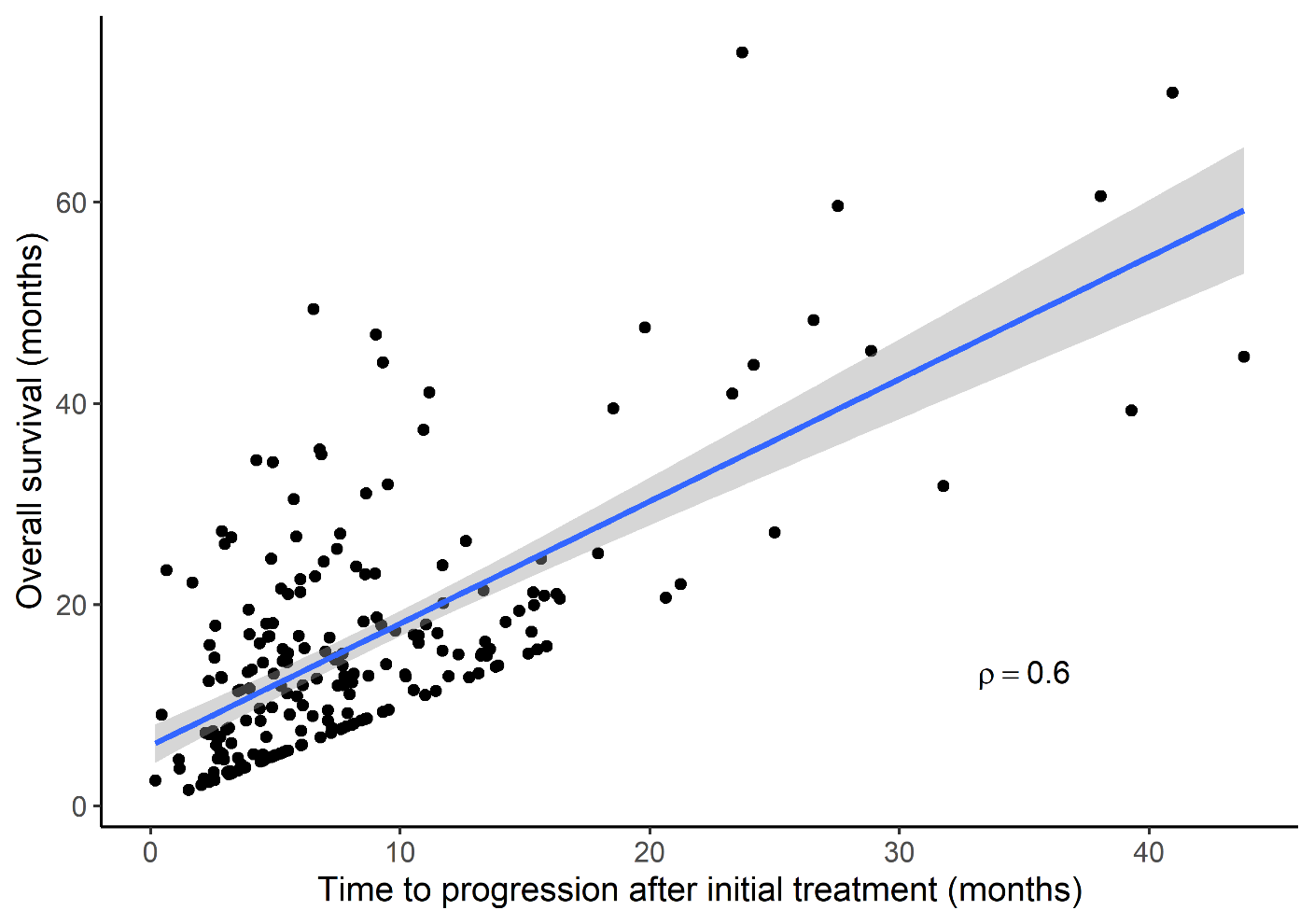


Figure S2. Correlation between time to progression after initial treatment and overall survival in The Cancer Genome Atlas glioblastoma dataset. Data are for 215 subjects with data on time to progression and overall survival. *P* = 3.15 x 10^-22^ for Spearman’s ρ. The line through the points is the linear regression line, and the shading represents the 95% confidence region for the regression line.
