# Supplementary material for: Gene signatures associated with prognosis and chemotherapy resistance in glioblastoma treated with temozolomide
Source: Front Genet. 2023 Dec 18;14:1320789. doi: 10.3389/fgene.2023.1320789 (PMC10802164; doi:10.3389/fgene.2023.1320789)
Supplement: Supplementary file 14 [file DataSheet1.DOCX]

Subjects in The Cancer Genome Atlas glioblastoma dataset

*N* = 606

No diagnosis of glioblastoma

*N* = 7

Primary diagnosis of glioblastoma

*N* = 599

Tumor tissue obtained from treated, primary glioblastoma

*N* = 54

Tumor tissue obtained from untreated, primary glioblastoma

*N* = 545

Did not receive concomitant or adjuvant temozolomide chemotherapy

*N* = 234

Received concomitant and/or adjuvant temozolomide chemotherapy

*N* = 311

No simple somatic variant data available from primary tumor

*N* = 89

Have data available on simple somatic variants from primary tumor

*N* = 222

No data available on vital status

*N* = 1

Have data available on vital status and overall survival

*N* = 221

Figure S1. Flow chart showing number of glioblastoma cases that were included in the analysis from The Cancer Genome Atlas database.
